# Effect of Ranitidine Intake on the Risk of Gastric Cancer Development

**DOI:** 10.3390/healthcare9081071

**Published:** 2021-08-20

**Authors:** SunMoon Kim, Suehyun Lee, JeeYoung Hong, Inseok Ko, Jong-Yeup Kim, Dong-Kyu Kim

**Affiliations:** 1 Division of Gastroenterology and Hepatology, Department of Internal Medicine, College of Medicine, Konyang University, Daejeon 35365, Korea; smkim@kyuh.ac.kr; 2 Department of Biomedical Informatics, College of Medicine, Konyang University, Daejeon 35365, Korea; shleemedi@kyuh.ac.kr (S.L.); sweetino@kyuh.ac.kr (I.K.); 3 Biomedical Research Institute, Konyang University Hospital, Daejeon 35365, Korea; jyhong@kyuh.ac.kr; 4 Department of Otorhinolaryngology-Head and Neck Surgery, College of Medicine, Konyang University, Daejeon 35365, Korea; 5 Department of Otorhinolaryngology-Head and Neck Surgery, Chuncheon Sacred Heart Hospital, Hallym University College of Medicine, Chuncheon 24253, Korea; 6 Institute of New Frontier Research, Hallym University College of Medicine, Chuncheon 24253, Korea

**Keywords:** East Asia, gastric cancer, histamine-2 blocker, *N*-nitrosodimethylamine, ranitidine

## Abstract

Gastric cancer is one of the most prevalent cancers globally, with high mortality, particularly in East Asia. Certain ranitidine products contain potentially carcinogenic *N*-nitrosodimethylamine. We investigated the potential association between gastric cancer risk and ranitidine intake using a nationwide cohort, extracted from the Korean National Health Insurance Service. In this longitudinal study, we employed a 1:1 propensity score matching according to sociodemographic factors. A total of 40,887 subjects were enrolled, of which 906 developed gastric cancer during the follow-up period. We investigated gastric cancer events during the follow-up period using the survival analysis, log-rank test, and Cox proportional hazards regression models to estimate incidence, survival rate, and hazard ratio. The incidence of gastric cancer was 67,422; 67,470; and 67,444 person-years in the control, other histamine-2 blockers, and ranitidine groups, respectively. Because the adjusted hazard ratio of gastric cancer was 0.98 and 1.01 in the other histamine-2 blockers and ranitidine groups, respectively, we could not calculate the likelihood of gastric cancer development in the ranitidine group. Ranitidine intake did not significantly increase the incidence of gastric cancer. Therefore, the relative risk of gastric cancer may be low in patients taking ranitidine products in South Korea.

## 1. Introduction

Gastric cancer is one of the most common cancers worldwide; however, its incidence is relatively high in Eastern and Central Asia and Latin America [1,2,3]. The majority of gastric cancers are adenocarcinomas, whereas gastrointestinal stromal tumors and primary gastric lymphoma account for a relatively low proportion of all gastric cancers. To date, well-known risk factors of gastric cancer include *Helicobacter pylori* infection, smoking, alcohol, and chemical exposure [4,5,6,7,8]. In addition, the findings of various studies support the hypothesis that the consumption of salted, smoked, and pickled foods containing high levels of nitrosamines might be associated with an increased risk of upper gastrointestinal tract cancer [9,10,11,12,13].

Nitrosamines are produced by the reaction of nitrates and nitrites to other proteins; *N*-nitrosodimethylamine (NDMA) is one of the most frequently occurring nitrosamines in various foods [14,15]. High concentrations of nitrosamines in the diet are carcinogenic [16,17]. In 2019, the US Food and Drug Administration announced that certain ranitidine medications were recalled because the NDMA levels in them were higher than the acceptable intake limit [18,19]. Following this, the Ministry of Food and Drug Safety in South Korea also suspended the manufacture and sale of 269 ranitidine products after assessing their NDMA levels. According to the ministry, seven of these products had NDMA levels as high as 53.50 ppm, which significantly exceeds the provisional standard of 0.16 ppm, assuming that a patient takes the maximal dose of 600 mg daily for life.

To date, several studies from other countries have noted that there is no demonstrable association between ranitidine long-term use and future gastric cancer [20,21,22]. However, other studies showed that NDMA-contaminated ranitidine could increase the risk of cancer [23,24]. Therefore, in this study, we investigated the association between the intake of ranitidine and the risk of gastric cancer development by comparing the incidence of gastric cancer in patients who were administered ranitidine versus that in patients who used other histamine-2 blockers or no treatment. For this analysis, we used a nationwide representative sample of 1,025,340 adults from the National Sample Cohort of the Korea National Health Insurance Service (KNHIS-NSC).

## 2. Experimental Section

### 2.1. Korea National Health Insurance Service

This study adhered to the tenets of the Declaration of Helsinki and used data from the national health claims database collected by the KNHIS. It was approved by the Institutional Review Board of Hallym Medical University, Chuncheon Sacred Hospital (No. 2020-52), and the need for written informed consent was waived as the KNHIS-NSC dataset used in the study comprised de-identified secondary data. The KNIHS employs the Korean Classification of Diseases (KCD), which is similar to the International Classification of Diseases, as a system of diagnostic practice codes. Here, we used data from the KNHIS-NSC collected from 2002 to 2013, containing information of 1,025,340 representative random subjects and accounting for approximately 2.2% of the South Korean population in 2002 (46 million). Stratified random sampling was performed using 1476 strata with respect to age (18 groups), sex (2 groups), and income level (41 groups: 40 with health insurance and 1 medical aid beneficiary).

### 2.2. Study Population

The patients in this study included all those who were prescribed ranitidine products (ranitidine and nizatidine) or other histamine-2 blockers (cimetidine, famotidine, roxatidine, and lafutidine) for more than 30 days between January 2002 and December 2008. Each patient was tracked until 2013, and patients diagnosed with gastric cancer (KCD C16) were identified. We excluded the following patients: (1) those under 20 years of age; (2) those who died as a result of any cause before 2009; (3) those diagnosed with any malignancy between 2002 and 2008; and (4) those diagnosed with other malignancies before the diagnosis of gastric cancer and several other gastric diseases (polyp, erosion, and ulcer: KCD D13.0, D13.1, D13.2, K25, and K26) since 2009. We also excluded patients who were prescribed proton-pump inhibitors (PPIs) because of their potential carcinogenicity in patients with gastric cancer arising from hypergastrinemia, gastric atrophy, and bacterial overgrowth in the stomach.

### 2.3. Predictor and Outcome Variables

The details of patients, including age, sex, residence, household income, disability, nonsteroidal anti-inflammatory drug (NSAID) doses, and history of smoking and alcohol consumption were obtained from the database. The study population was divided into three age groups (<45, 45–64, and >65 years), three income groups (low: ≤30%, middle: 30.1–69.9%, and high: ≥70% of the median), three residential areas (Seoul, the largest metropolitan region in South Korea; other metropolitan cities in South Korea; and small cities and rural areas), and two disability-based groups (with and without). In addition, five groups were formed according to NSAID dose (none, ≤60 days, 61–120 days, 121–180 days, and >180 days) and three groups according to smoking history (never, former, and current) and alcohol use (rare; intermediate: 1–2 times per month/week and <3 shots of soju; heavy: ≥3 times per week and >7 shots of soju). Soju is a clear, colorless distilled beverage of Korean origin, and a shot of soju contains 11 g of alcohol. The operational definitions of the study endpoints were all-cause mortality or gastric cancer incidence. All patients who had no event and were alive until 31 December 2013 were censored after this time point. The risk of gastric cancer was compared between the ranitidine group, other histamine-2 blockers group, and the control group using person-years at risk, which was defined as the duration between the start of ranitidine or other histamine-2 blockers or 1 January 2009 (for the control) and their respective endpoints.

### 2.4. Statistical Analysis

We employed 1:1 propensity score-matching according to age, sex, residential area, household income, disability, NSAID dose, and history of smoking and alcohol use. The incidence rate of gastric cancer per 1000 person-years was obtained by dividing the number of patients with gastric cancer by person-years at risk. The overall disease-free survival rate was determined using the Kaplan–Meier survival curves throughout the observation period. To identify whether ranitidine or other histamine-2 blockers increased the risk of gastric cancer development, we used the Cox proportional hazard regression model to calculate the hazard ratio (HR) and 95% confidence intervals (CI), adjusting for other predictor variables. All statistical analyses were performed using R version 3.3.1 (R Foundation for Statistical Computing, Vienna, Austria) with a significance level of 0.05.

## 3. Results

### 3.1. Incidence of Gastric Cancer

The study population comprised 13,629 subjects divided into three groups: control, other histamine-2 blockers, and ranitidine (Table 1). There was no significant difference in the demographic variables among these three groups, indicating an appropriate matching of groups. A total of 67,422; 67,470; and 67,444 person-years were evaluated in the control, other histamine-2 blockers, and ranitidine groups, respectively (Table 2), and the incidence rate of gastric cancer was similar among these groups. However, a higher incidence of gastric cancer was observed in males (vs. females), old subjects (vs. young and middle-aged), rural area residents (vs. urban residents), subjects with a low household income (vs. high household income), those with a disability (vs. none), those taking NSAIDs for >20 days (vs. no NSAID), smokers (vs. non-smokers), and subjects with rare alcohol consumption (vs. heavy alcohol consumption).

### 3.2. Hazard Ratio

**Figure 1:**
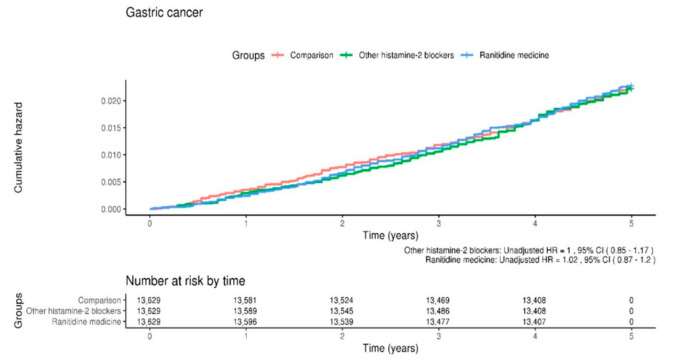
Kaplan–Meier survival curves and log-rank tests for the development of gastric cancer in the three study groups: control, other histamine-2 blockers, and ranitidine.

We used univariate and multiple Cox regression models to analyze the HR for the development of gastric cancer (Table 3). After adjusting for sociodemographic factors, the ranitidine group was found to be associated with prospective gastric cancer development with an adjusted HR of 1.01 (95% CI, 0.86–1.18), whereas the other histamine-2 blockers showed subsequent gastric cancer development with an adjusted HR of 0.98 (95% CI, 0.84–1.15). Thus, no significant difference was found among the three groups in terms of subsequent gastric cancer development. The Kaplan–Meier survival curves using the log-rank tests for gastric cancer-free survival rate revealed no significant difference in the overall disease-free survival rates for gastric cancer among the three groups (Figure 1).

### 3.3. Predictor and Outcome Variables

We observed a significant likelihood of gastric cancer development in males (adjusted HR of 1.88; 95% CI, 1.6–2.22), patients > 65 years of age (39.05; 31.64–48.19), rural area residents (1.33; 1.09–1.61), subjects with a disability (1.93; 1.53–2.44), those taking NSAIDs for > 120 days (1.64; 1.19–2.25 in 121–180 days and 1.24; 0.96–21.62 in > 180 days), and smokers (1.39; 1.17–1.67). Subjects with a high household income showed a significantly low rate of gastric cancer development (0.75; 0.63–0.88).

## 4. Discussion

Ranitidine is widely used to treat gastrointestinal problems such as heartburn and acid indigestion. However, in 2019, authorities from the USA and South Korea recommended to recall certain ranitidine products owing to contamination with NDMA, designated by the World Health Organization’s International Cancer Research Institute as a potentially carcinogenic substance to humans, categorized in Group 2A [25]. In this longitudinal study, we investigated the association between the intake of ranitidine and the prospective risk of gastric cancer using the KNHIS-NSC dataset. Consistent with previous studies, we found no significant difference in terms of gastric cancer development among the three study groups (control, other histamine-2 blockers, and ranitidine) [20,21,22]. We observed a significant likelihood of gastric cancer development in males, patients > 65 years of age, rural area residents, subjects with a disability, those taking an NSAID for >120 days, and smokers, while a low risk of gastric cancer development was observed in individuals with a high household income.

We sociodemographically matched individuals from a nationwide cohort database of 1,025,340 South Koreans. Interestingly, when we compared the ranitidine group with the control or other histamine-2 blocker groups, we found a similar incidence of gastric cancer among all the three groups (4.40 in the control group, 4.40 in the other histamine-2 blockers group, and 4.50 in the ranitidine group). Additionally, the adjusted HR for gastric cancer in the ranitidine group was not significant (adjusted HR = 1.01, 95% CI = 0.86–1.18), as indicated by the results of the multiple Cox regression analyses of all variables. Moreover, the Kaplan–Meier survival curves with the log-rank test results indicated no significant difference in gastric cancer-free survival rates among the three groups. These findings suggest that the intake of ranitidine, potentially containing NDMA, may not contribute to the development of gastric cancer in South Koreans.

This study has some unique strengths. Firstly, this is the first nationwide, population-based retrospective cohort study to evaluate the incidence of gastric cancer in patients using ranitidine by means of the KNHIS-NSC dataset, which proved to be reliable [26,27,28,29]. Additionally, the data we extracted from the KNHIS database are based on the entire Korean population, thereby eliminating any selection bias. Secondly, to improve the validity of our results, we matched almost all confounding factors related to gastric cancer, including sex, age, history of smoking and alcohol use, and NSAID dosage, excluding patients using PPIs. PPIs are widely prescribed to suppress gastric acid production. Hence, the resulting hypergastrinemia, gastric atrophy, and bacterial overgrowth in the stomach are reported to result in carcinogenicity in patients with gastric cancer [30,31,32]. Finally, we analyzed the adjusted HRs of gastric cancer development in participants administered other histamine-2 blockers and compared them with those in the ranitidine group. Histamine-2 blockers such as cimetidine, famotidine, nizatidine, and ranitidine are prescribed to reduce the amount of acid produced by the cells in the stomach lining. Thus, we compared ranitidine with other histamine-2 blockers in connection with the risk of gastric cancer development.

Our study also had some limitations. First, during propensity score matching, we did not consider *H. pylori* infection, which accounts for the majority of non-cardia gastric cancers by triggering gastric inflammation and the subsequent neoplastic progression [33,34,35]. Thus, its eradication can reduce the subsequent risk of developing gastric cancer. However, we could not access pathology reports to determine whether *H. pylori* infection was present in our study participants. Second, we were unable to access other specific health data, including body mass index, lipid profiles, and behavioral risk factors such as chemical exposure. Therefore, these possible confounding factors could not be controlled in this study. Third, inherited mutations of certain genes such as *glutathione S-transferase mu 1*-null phenotype and *cadherin 1* are known risk factors for gastric cancer and contribute to 1–3% of all gastric cancers [2]. However, the KNHIS-NSC did not include any genetic information. Fourth, this study had a 5-year follow-up period, which may not be long enough to detect gastric cancer development. Finally, mortality-related data were not available in our registry.

In conclusion, we investigated the possible link between the intake of ranitidine and the prospective development of gastric cancer. We found no significant difference in terms of gastric cancer development among the three study groups (control, other histamine-2 blockers, and ranitidine), suggesting that the intake of ranitidine, even if it contains NDMA, may not be associated with an increased risk of developing gastric cancer.

## Figures and Tables

**Table 1:** Characteristics of the study participants.

Variable	Comparison (*n* = 13,629)	Other Histamine-2 Blockers (*n* = 13,629)	Ranitidine Medicine (*n* = 132,629)	*p* Value
Sex				0.976
Female	5848 (42.9%)	5830 (42.8%)	5838 (42.8%)	
Male	7781 (57.1%)	7799 (57.2%)	7791 (57.2%)	
**Age (years)**				>0.999
20–44	9204 (67.5%)	9207 (67.6%)	9211 (67.6%)	
45–64	3740 (27.4%)	3741 (27.4%)	3739 (27.4%)	
≥65	685 (5.0%)	681 (5.0%)	679 (5.0%)	
**Residence**				>0.999
Seoul (metropolitan)	2884 (21.2%)	2880 (21.1%)	2891 (21.2%)	
Second area (other metropolitan)	3513 (25.8%)	3517 (25.8%)	3518 (25.8%)	
Third area	7232 (53.1%)	7232 (53.1%)	7220 (53.0%)	
**Household income**				0.999
Low (0–30%)	3213 (23.6%)	3209 (23.5%)	3221 (23.6%)	
Middle (31–70%)	5395 (39.6%)	5379 (39.5%)	5377 (39.5%)	
High (71–100%)	5021 (36.8%)	5041 (37.0%)	5031 (36.9%)	
**Disability**				0.539
No	13,238 (97.1%)	13,219 (97.0%)	13,207 (96.9%)	
Yes	391 (2.9%)	410 (3.0%)	422 (3.1%)	
**NSAID**				>0.999
No	1700 (12.5%)	1697 (12.5%)	1700 (12.5%)	
<60 days	8082 (59.3%)	8088 (59.3%)	8080 (59.3%)	
60–120 days	1883 (13.8%)	1872 (13.7%)	1872 (13.7%)	
120–180 days	546 (4.0%)	559 (4.1%)	561 (4.1%)	
>180 days	1418 (10.4%)	1413 (10.4%)	1416 (10.4%)	
**Smoking**				>0.999
Never	8671 (63.6%)	8664 (63.6%)	8661 (63.5%)	
Former	1491 (10.9%)	1502 (11.0%)	1500 (11.0%)	
Current	3467 (25.4%)	3463 (25.4%)	3468 (25.4%)	
**Alcohol Drinking habit**				>0.999
Rare	7258 (53.3%)	7252 (53.2%)	7240 (53.1%)	
Intermediate	5222 (38.3%)	5228 (38.4%)	5235 (38.4%)	
Heavy	1149 (8.4%)	1149 (8.4%)	1154 (8.5%)	

**Table 2:** Incidence of gastric cancer according to different variables.

Variable	Case	Person Year	Incidence
**Group**			
Control	300	67,422	4.40
Other histamine-2 blockers	300	67,470	4.40
Ranitidine medicine	306	67,444	4.50
**Sex**			
Female	291	86,930	3.30
Male	615	115,406	5.30
**Age (years)**			
20–44	151	137,703	1.10
45–64	365	55,254	6.60
≥65	390	9380	41.60
**Residence**			
Seoul (metropolitan)	128	42,954	3.00
Second area (other metropolitan)	192	52,312	3.70
Third area	586	107,070	5.50
**Household income**			
Low (0–30%)	275	47,602	5.80
Middle (31–70%)	330	80,003	4.10
High (71–100%)	301	74,731	4.00
**Disability**			
No	826	196,401	4.20
Yes	80	5935	13.50
**NSAID**			
No	78	25,310	3.10
<60 days	369	120,330	3.10
60–120 days	122	27,853	4.40
121–180 days	77	8169	9.40
>180 days	260	20,673	12.60
**Smoking**			
Never	576	128,655	4.50
Former	94	22,240	4.20
Current	236	51,441	4.60
**Alcohol drinking habit**			
Rare	578	107,445.2	5.40
Intermediate	245	77,839.9	3.10
Heavy	83	17,050.6	4.90

**Table 3:** Hazard ratio (95% confidence interval) of gastric cancer according to different variables.

Variable	Unadjusted HR ^1^ (95% CI)	*p* Value	Adjusted HR ^1^ (95% CI)	*p* Value
**Group**				
Control	1 (ref)		1 (ref)	
Other histamine-2 blockers	1 (0.85–1.17)	0.99	0.98 (0.84–1.15)	0.83
Ranitidine medicine	1.02 (0.87–1.2)	0.81	1.01 (0.86–1.18)	0.94
**Sex**				
Female	1 (ref)		1 (ref)	
Male	1.59 (1.39–1.83)	0.00	1.88 (1.6–2.22)	0.00
**Age** (**years**)				
20–44	1 (ref)		1 (ref)	
45–64	6.04 (4.99–7.3)	0.00	5.94 (4.88–7.23)	0.00
≥65	38.5 (31.9–46.45)	0.00	39.05 (31.64–48.19)	0.00
**Residence**				
Seoul (metropolitan)	1 (ref)		1 (ref)	
Second area (other metropolitan)	1.23 (0.98–1.54)	0.07	1.1 (0.88–1.38)	0.39
Third area	1.84 (1.52–2.23)	0.00	1.33 (1.09–1.61)	0.00
**Household income**				
Low (0–30%)	1 (ref)		1 (ref)	
Middle (31–70%)	0.71 (0.61–0.84)	0.00	1.01 (0.86–1.19)	0.90
High (71–100%)	0.7 (0.59–0.82)	0.00	0.75 (0.63–0.88)	0.00
**Disability**				
No	1 (ref)		1 (ref)	
Yes	3.22 (2.56–4.05)	0.00	1.93 (1.53–2.44)	0.00
**NSAID**				
No	1 (ref)		1 (ref)	
<60 days	1 (0.78–1.27)	0.97	1.02 (0.8–1.31)	0.86
60–120 days	1.42 (1.07–1.89)	0.02	1.04 (0.78–1.38)	0.81
121–180 days	3.07 (2.24–4.2)	0.00	1.64 (1.19–2.25)	0.00
>180 days	4.09 (3.18–5.27)	0.00	1.24 (0.96–1.62)	0.10
**Smoking**				
Never	1 (ref)		1 (ref)	
Former	0.94 (0.76–1.17)	0.61	0.99 (0.78–1.25)	0.92
Current	1.02 (0.88–1.19)	0.75	1.39 (1.17–1.67)	0.00
**Alcohol drinking habit**				
Rare	1 (ref)		1 (ref)	
Intermediate	0.58 (0.50–0.68)	0.00	0.84 (0.72–1.00)	0.05
Heavy	0.9 (0.72–1.14)	0.39	1.18 (0.92–1.52)	0.19

^1^ HR = hazard ratio; CI = confidence interval.

## Data Availability

The authors confirm that the data supporting the findings of this study are available within the article.
